# Serum levels of MMP-11 correlate with clinical outcome in Chinese patients with advanced gastric adenocarcinoma

**DOI:** 10.1186/1471-2407-11-151

**Published:** 2011-04-24

**Authors:** Dong Yan, Hong Dai, Jin-Wei Liu

**Affiliations:** 1 Department of Oncology, Beijing Chao-Yang Hospital affiliated with Capital Medical University, 8# Baijiazhuang Road, Beijing, China; 2 Department of Clinical Chemical Examination, Beijing Chao-Yang Hospital affiliated with Capital Medical University, 8# Baijiazhuang Road, Beijing, China

**Keywords:** advanced gastric adenocarcinoma, Matrix metalloproteinase 11, overall survival, prognosis, TTP

## Abstract

**Background:**

The aim of the present study was to investigate the association between serum levels of matrix metalloproteinase 11 (MMP-11) and responses to front-line chemotherapy and prognosis in advanced unresectable gastric adenocarcinoma.

**Methods:**

Clinical data concerning 86 patients with advanced gastric adenocarcinoma (stages III c to IV), treated in Beijing Chao-Yang Hospital from 2005 to 2009, were reviewed retrospectively. Adenocarcinoma was confirmed by pathology and patients received 5-fluorouracil-based front-line combination chemotherapy with third generation chemotherapeutic agents including paclitaxel, docetaxel and oxaliplatin. The regimen was repeated every two to three weeks, and the first evaluation was carried out after three cycles. The median cycle of chemotherapy was 6 (ranging from three to twelve cycles). Serum MMP-11 protein from the 86 patients was examined using enzyme-linked-immunosorbent-assay (ELISA) prior to chemotherapy and after three cycles of chemotherapy. Serum samples from healthy individuals were used as controls.

**Results:**

The response rate (RR, complete response plus partial response) to chemotherapy in the 86 patients was 44.2% (38/86). The median TTP (time to progression) and overall survival (OS) in patients who responded to chemotherapy were 6.0 and 10.0 months, respectively. The response rate to chemotherapy in patients with high levels of serum MMP-11 (42.9%; 9/21) was similar to that in patients with low levels (44.6%; 29/65) (P = 0.935). Patients with low serum levels of MMP-11 had a higher median survival time and 1-year survival rate than those with high levels (11 months vs. 8 months, 50.2% vs. 21.7%, P = 0.017), although the TTP was comparable in all patients, irrespective of serum MMP-11 level (P = 0.178). Serum MMP-11 levels were correlated with lymph node metastasis (P = 0.006). Cox multivariate regression analysis demonstrated that the serum level of MMP-11 was an independent prognostic factor for patients presenting with advanced gastric carcinoma.

**Conclusions:**

Serum levels of MMP-11 in Chinese patients with advanced gastric carcinoma were not associated with the response to front-line chemotherapy, but could play an important role in lymph node metastasis and prognosis.

## Background

Gastric cancer (GC) is the second most common cancer world-wide, and the third leading cause of cancer-related deaths in China. Although the most effective treatment for GC is surgical resection, approximately 70% of patients presenting with GC have a locally advanced and metastatic disease at the time of initial diagnosis, precluding the option of surgical resection [1]. Therefore, systemic cytotoxic chemotherapy is a major therapeutic strategy for advanced gastric carcinoma. The combination of third generation chemotherapeutic agents including paclitaxel, docetaxel and oxaliplatin with 5-fluorouracil have improved therapeutic response rates and overall survival by approximately 20-30% and 4-6 months, respectively. However, this treatment can result in clinically significant adverse effects. In order to improve survival outcome and administer the most effective treatment, there is a requirement for more sensitive tumor markers than those currently available.

Matrix metalloproteinases (MMPs) are a family of zinc-dependent endopeptidases that mediate degradation of components of the extracellular matrix [2]. They are predominantly produced by stromal cells in response to the presence of tumor cells [3]. MMP-11 is a member of the MMP family that degrades ECM components and may play a central role in the enhancement of tumor-induced angiogenesis, cell migration, proliferation, apoptosis and connective tissue degradation [4,5]. MMP-11 differs from other MMPs, which are expressed as proenzymes and processed to active forms through proteolytic cleavage activated extracellularly, indicating that MMP-11 could have a unique role in tumor development and progression [6]. Clinicopathological studies have demonstrated that MMP-11 is an important factor in tumor progression in various malignant tumors including pulmonary cancer [7], head and neck carcinoma [8,9] and breast carcinoma [10].

Previously, we demonstrated that MMP-11 serum levels were significantly higher in patients suffering from GC than in healthy individuals, and were correlated with recurrence of GC. Zhao et al [11] demonstrated that MMP-11 expression was associated with advanced-stage and high-grade tumors, and correlated with increased expression of IGF-1. Min et al [12] suggested that IGF-1 receptor blockade could increase radiation and chemotherapy-induced apoptosis in mice. The aim of the present study was to investigate whether elevated MMP-11 expression could predict responses to front-line chemotherapy and prognosis of Chinese patients with advanced gastric carcinoma.

## Methods

### Patient Selection

Eighty-six patients with histopathologically confirmed advanced unresectable gastric adenocarcinoma (stages III c to IV) from March 2005 to February 2009 in Beijing Chao-Yang Hospital were studied retrospectively. Eligibility for the study required patients to have an Eastern Cooperative Oncology Group performance status of 0 to 2, and available plasma and measurable tumor focus evaluated by multi-detector spiral CT scanning. Patients had received front-line chemotherapy for at least three cycles and were observed for chemotherapy responses and survival outcomes. Tumors were staged according to the criteria of the American Joint Committee on Cancer (AJCC) TNM stage classification, sixth edition (2002) for Gastric Cancer. In accordance with the RECIST guidelines [13], response to therapy was categorized into four groups: complete response (CR), partial response (PR), stable disease (SD) and progression of disease (PD), with CR and PR confirmed for four weeks. For tumor response assessment, objective responses after three cycles of treatment were evaluated on the basis of computed tomography (CT) scans. Overall survival was calculated as the time from the beginning of chemotherapy to death or the last follow-up. The time to progression of disease was calculated from the date that chemotherapy began to the date of tumor progression. Twenty healthy volunteers were recruited as a control group. There was no evidence of malignancy on abdominal computed tomography examination of these individuals, and results of physical and laboratory examinations were normal. Individuals were excluded from the study if they did not give consent.

### Specimen Collection

A total of 86 serum samples from patients with advanced gastric carcinoma and 20 samples from healthy individuals were obtained from the clinical laboratory. Serum samples from patients were collected in a fasting state early in the morning prior to front-line chemotherapy and after three cycles. Venous blood samples were collected in plain tubes, allowed to clot for one hour and centrifuged twice (2500 g) for 10 minutes at 4°C to obtain serum. The serum was removed, aliquoted and stored at -70°C until required.

### MMP-11 Assay

The concentration of MMP-11 protein was measured with a commercially available sandwich enzyme-linked immunoadsorbent assay kit based on monoclonal antibodies (Adlitteram Diagnostic Laboratories. Inc). Each sample was measured in duplicate. Serum samples from patients and control subjects were incubated in microtiter plates. Distilled or deionized water was added to the plates to be used as a blank control. Enzyme conjugate reagent (50 μl) was added to each well, incubated for 60 min at 37°C and washed 5 times with distilled or deionized water. Finally, 50 μl of color A and B reagents were added to each well and incubated for 15 min. Stop solution (50 μl) was placed in each well and mixed for 30 s. The color development was measured by absorbance at 450 nm using a microtiter plate reader. Accurate sample concentrations of MMP-11 were determined by comparing the specific absorbance with those obtained from the standards plotted on a standard curve.

### Statistical analysis

Statistical analysis was carried out using the Chi-square test, Fisher's exact test, ANOVA and independent-samples t test. For all analyses, p < 0.05 was considered statistically significant. The 95% CI for hazard ratios and frequencies were calculated as exact CI. Receiver operating characteristic (ROC) curves were constructed by plotting sensitivity vs. (1-specificity), and the areas under the ROC curves (AUCs) were calculated. Kaplan-Meier curves were compared for survival using the standard log-rank test. To make further analyses with Kaplan-Meier curves possible, patients were split into two groups, one comprising patients with elevated MMP-11 protein and the other comprising patients without elevated MMP-11. The cut-off for this categorized variable was set at the 75th percentile of MMP-11 in the total group. SPSS software (version 15) was used for statistical analysis.

## Results

### Patient Characteristics

Eighty-six patients with advanced gastric carcinoma (stages III c to IV) who received palliative chemotherapy were included in the current analyses. Among these patients, 60 were male and 26 were female. The median age was 51 years (ranging from 50-56 years). Twenty three patients had well differentiated adenocarcinoma, 63 presented with poorly differentiated adenocarcinoma. Histological differentiation determined that 54 patients (62.8%) had intestinal type adenocarcinoma, 20 (23.2%) had mucinous carcinoma and signet-ring cell carcinoma was present in 12 patients (14.0%). Clinical details are listed in Table 1. In the control group, 15 individuals were male and five were female. The median age was 40 years (ranging from 37-42 years).

**Table 1:** MMP-11 Levels and Clinical Characteristics

Clinical data	No. of Case (%)	MMP-11 level	**P **^**-value**^
			
		Mean ± SD (ng/ml)	
NO. of patients	86		

Gender			
Male	60(69.8%)	45.76 ± 22.89	0.200
Female	26(30.2%)	38.79 ± 23.17	

Age (yrs)			
Median	51(50-56)		
<65	63(73.3%)	43.40 ± 22.76	0.166
≥65	23(26.7%)	50.79 ± 21.96	

Differentiation			
G1, G2	23(26.7%)	48.56 ± 23.30	0.235
G3	63(73.3%)	41.86 ± 22.90	

Histology			
Intestinal type adenocarcinoma	54(62.8%)	44.22 ± 23.92	
Mucinous carcinoma	20(23.2%)	45.89 ± 17.99	0.527
Signet-ring cell carcinoma	12(14.0%)	53.44 ± 23.80	

ECOG			
0-1	68(79.1%)	46.14 ± 21.40	0.610
2	18(20.9%)	43.33 ± 26.49	

Disease stage			
III c	26(30.2%)	43.22 ± 31.02	0.288
IV	60(69.8%)	48.45 ± 23.57	

Metastasis site			
Lymph node	35(40.7%) ^*#^	52.55 ± 24.72	
Peritoneum	21(24.4%) ^#^	33.44 ± 14.46	0.006 (*0.029, ^#^0.002)
Internal organs	30(34.9%) *	40.42 ± 22.74	

Chemotherapy cycles	377		
Median	6.00(3.00-12.00)		

mTTP (m)			
Median	6.00(5.29-6.71)		

mOS (m)			
Median	10.00(8.51-11.50)		

Response			
PR	38(44.2% )	46.85 ± 24.92	0.182
SD	22(25.6% )	37.34 ± 19.95	
PD	26(30.2% )	48.97 ± 20.23	

Abbreviations: PR, partial response; SD, stable disease; PD, progression of disease. Analysis of the difference between groups using analysis of variance (ANOVA). Where differences were identified, further analysis (between pairs of groups, with respective pairs for each molecule marked with *and *) was performed using Scheffe (95% confidence interval (CI))

### MMP-11 protein levels in sera of gastric carcinoma

**Figure 1:**
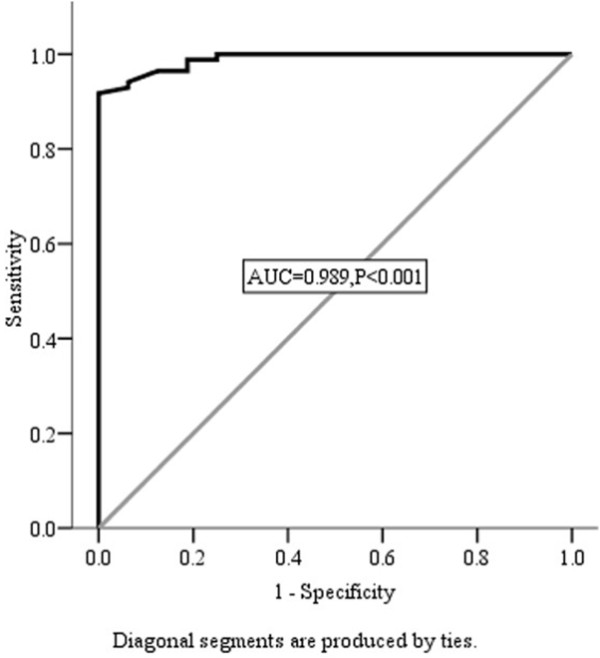
**Serum MMP-11 ROC curve**. Receiver operating characteristic (ROC) curve of serum MMP-11 concentration: AUC = 0.989, P < 0.001.

The serum levels of MMP-11 protein were significantly higher in patients with advanced gastric adenocarcinoma (n = 86; median = 41.74 ng/ml; range from 10.43 to 97.37 ng/ml) than in controls (n = 20; median = 6.74 ng/ml; range from 5.71 to 8.56 ng/ml; p < 0.001). Using ROC analysis, a cut-off level of MMP-11 protein at 14.38 ng/ml was associated with optimal sensitivity and specificity of 94.1% and 93.7%, respectively, for diagnosis. ROC curve analysis is presented in Figure 1.

### Correlation between MMP-11 protein levels and clinicopathological characteristics

The relationship between MMP-11 protein levels and patient clinicopathological parameters is summarized in Table 1. Serum levels of MMP-11 protein were not correlated with patient age, sex, disease stage and grade of differentiation. However, MMP-11 protein levels were significantly higher in patients suffering with lymph node metastasis, including 31 local and 4 distant lymph node metastases, compared with peritoneal seeding and internal organ metastasis (P = 0.002 and P = 0.029, respectively).

### Correlation between MMP-11 protein levels and chemotherapy response

All patients had measurable tumor foci and received 5-fluorouracil-based combination chemotherapy for at least three cycles (Table 2). PR was achieved in 44.2% (38/86) of patients, 25.6% (22/86) had SD and 30.2% (26/86) experienced PD with a median duration of six months and overall survival of ten months. MMP-11 serum levels were not correlated with the front-line chemotherapy response (P = 0.182). Patients were divided into two groups according to MMP-11 levels (75th percentile or higher, and lower than the 75th percentile). A total of 21 patients (24.4%) had higher MMP-11 levels than the 62.09 ng/ml cut-off. Of these patients, 42.9% (9/21) achieved PR, 28.6% (6/21) had SD and 28.5% (6/21) experienced PD. These results were similar to the 44.6% (29/65) PR, 24.6% (16/65) SD and 30.8% (20/65) PD evident in the low MMP-11 level group (P = 0.935). MMP-11 levels were differently altered in PR and PD. MMP-11 protein was lower than baseline in patients demonstrating PR (median = 45.15 ng/ml, 95%CI: 35.79-62.09 vs. 44.12 ng/ml, 95%CI: 34.32-56.52; P = 0.147). However, it showed an elevated trend in those with PD (median = 47.29 ng/ml, 95%CI: 36.05-53.73 vs. 50.52 ng/ml, 95%CI: 37.74-54.22; P = 0.297).

**Table 2:** Chemotherapy Regimens Used in AGC

Chemotherapy regimen	Number of patients
Paclitaxel, cisplatin, 5-fluorouracil	37
Paclitaxel, 5-fluorouracil	21
Oxaliplatin, 5-fluorouracil	18
Docetaxel, 5-fluorouracil	10

### Correlation between MMP-11 levels and survival

**Figure 2:**
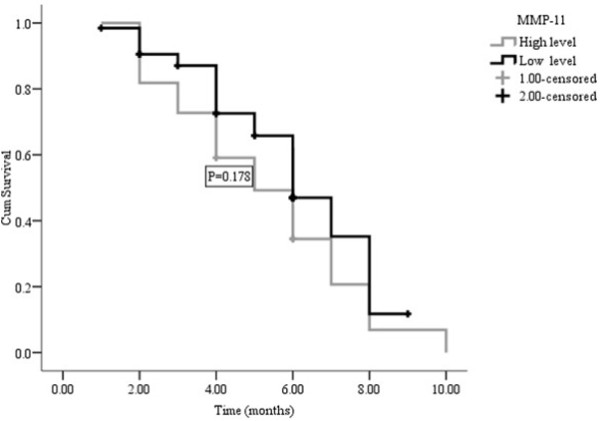
**TTP curves of high level MMP-11 compared with low level MMP-11**.

**Figure 3:**
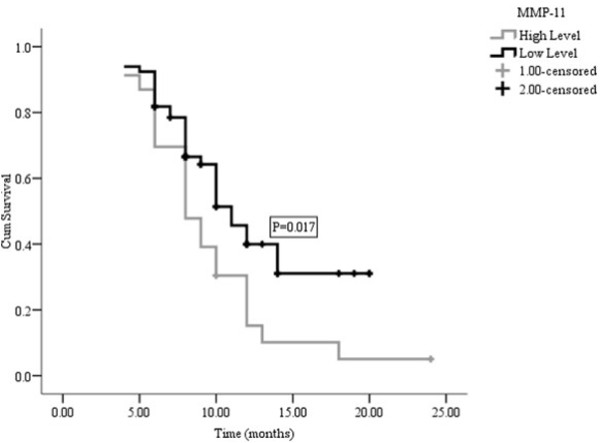
**Overall survival curves for high level MMP-11 compared with low level MMP-11**.

The median follow-up time was 10.50 months (ranging from 9.07 to 12.94 months); the median TTP, OS and 1-year survival were six months, ten months and 40.0%, respectively. The median TTP time for patients with low levels of MMP-11 was not significantly different from patients with high levels (median = 6.0, range from 4.98 to 7.02 months vs. median = 5.0 months, range from 3.28 to 6.72 months; P = 0.178, Figure 2). However, patients with low level MMP-11 protein had an higher median survival time and 1-year survival rate than those with high levels (11 vs. 8 months, 50.2% vs. 21.7%, P = 0.017, Figure 3) in a multivariate analysis that considered MMP-11 level as an independent factor of OS in advanced gastric carcinoma (HR = 2.618, 95%CI 1.288-5.320, P = 0.018, Table 3).

**Table 3:** Association between Clinical Features and Survival Using Multivariate Analysis

Parameter		Multivariate Analysis		
		
		**P **^**-value**^	HR	95%CI
Gender	Male	0.622	1.199	0.582-2.473
	Female		1	
Age	<65	0.215	1.569	0.770-3.199
	≥65		1	
Histology	Intestinal type adenocarcinoma	0.150	0.415	0.126-1.373
	Mucinous carcinoma	0.588	0.752	0.268-2.106
	Signet-ring cell		1	
Differentiation	G1, G2	0.492	0.761	0.350-1.658
	G3		1	
Metastatic site	Dissemination	0.516	1.387	0.578-3.437
	Local		1	
Stage of disease	III c	0.025	0.273	0.088-0.849
	IV		1	
MMP-11 protein level	High level	0.018	2.618	1.288-5.320
	Low level		1	

Abbreviation: HR, hazard ratio; CI, confidence interval.

## Discussion

This study demonstrated that MMP-11 over-expression was consistently detected in advanced gastric adenocarcinoma. MMP-11 serum levels in 86 cases of advanced gastric adenocarcinoma were not associated with responses to 5-fluorouracil-containing chemotherapy and TTP as front-line chemotherapy. However, they correlated with lymph node status and mOS. These data suggest that MMP-11 could be involved in metastatic dissemination and be regarded as an independent prognostic factor of advanced gastric adenocarcinoma.

Despite improvements in surgical techniques and the development of new chemotherapy regimes, gastric carcinoma remains the third leading cause of cancer-related deaths in China [14]. Previously, we demonstrated that MMP-11 was a cancer-related protein and its over-expression was consistently confirmed at mRNA and protein levels in primary gastric adenocarcinoma tumors compared with matched normal tissues. Currently, the impact of MMP-11 on advanced gastric adenocarcinoma is uncertain. The aim of the study was to evaluate the associations between the expression of MMP-11 protein in serum and response to front-line chemotherapy and prognosis in advanced gastric adenocarcinoma. The results demonstrate that serum MMP-11 levels are not correlated with response to front-line chemotherapy, but are correlated with lymph node status and mOS in patients with advanced gastric adenocarcinoma.

MMPs have a broad spectrum of proteolytic activities towards extracellular matrix (ECM) components, and are believed to be involved in several biological processes including embryo implantation and morphogenesis, cell migration, metastasis, tumor invasion and wound healing [15,16]. MMP-11 over-expression has been demonstrated in human cancers including oral cancer [17,18], desmoid tumors [19], non-small cell lung cancer [20] and esophageal adenocarcinoma [21]. Furthermore, increased MMP-11 gene expression is correlated with increased aggressiveness of tumors and a poor clinical outcome [22,23]. Clinical trials have demonstrated that a high level of MMP-11 expression is correlated with a lower survival rate among patients with breast, non-small cell lung cancer and colon cancer [24,25]. Deng et al [6] reported that the BGC823 cell-line silenced MMP-11 expression and significantly inhibited cell proliferation and colony formation in soft agar medium and tumorigenicity in nude mice. The same authors also demonstrated that MMP-11 promoted the proliferation, invasion and tumorigenicity of the AGS cell line. Such experimental evidence has provided a causative role for MMP-11 protein during tumor progression. This study is the first to identify a correlation between MMP-11 expression and outcome in advanced gastric adenocarcinoma patients. In this study, MMP-11 protein levels appeared to correlate with invasion potential and high levels were related to the extent of lymph node metastasis in GC (P = 0.006), similar to data reported for other malignancies [26]. Moreover, this study demonstrates that patients with high level MMP-11 expression had a shorter survival time than patients with low levels of MMP-11. In other words, the findings indicate that MMP-11 protein is an independent prognostic factor for patients with advanced gastric adenocarcinoma. As has been reported previously in breast and head and neck cancer, there were no obvious associations among the degree of tumor differentiation, MMP-11 protein level, sex and age.

There was no statistical difference between MMP-11 levels with mTTP and chemotherapy response. Potential reasons for this could be: (1) there were no differences in terms of the effects of MMP-11 on mTTP and chemotherapy response in advanced gastric adenocarcinoma; (2) the number of cases studied in the current research was not large enough. The OS in the present study was different from the results reported for the V325 trial [27]. Possible explanations include the fact that this was a retrospective study; several factors could influence survival. The chemotherapy regimens used in the present study differed in some ways from that used in the V325 trial, which could affect OS.

## Conclusions

This study suggests that serum levels of MMP-11 protein in Chinese patients with advanced GC are not associated with responses to front-line chemotherapy. However, MMP-11 could have a role in lymph node metastasis and could potentially be an independent factor for prognosis.

## Competing interests

I certify that my affiliations with, or financial involvement in, any organization or entity with a financial interest in or financial conflict with the subject matter or materials discussed in this manuscript, within the past five years and foreseeable future, are disclosed.

## Authors' contributions

Dong Yan analyzed and interpreted MMP-11 expression in advanced gastric adenocarcinoma, and was a major contributor to writing of the manuscript. Jin-Wei Liu performed the examination of MMP-11. All authors read and approved the final manuscript.

## Pre-publication history

The pre-publication history for this paper can be accessed here:

http://www.biomedcentral.com/1471-2407/11/151/prepub
